# OP15 Cost-Effectiveness Of Quantitative Fecal Immunochemical Tests For Detecting Suspected Colorectal Cancer In The Context Of Colonoscopy Capacity Constraints

**DOI:** 10.1017/S0266462324000783

**Published:** 2025-01-07

**Authors:** Aline Navega Biz, Matt Stevenson, Sophie Whyte, Jean Hamilton, Shijie Ren, Laura Heathcote, Emma Simpson, Katy Cooper, Mark Clowes, Alex Ball, Sally Benton, Rachel Carten, Matt Kurien, Kevin Monahan, Sue Harnan

## Abstract

**Introduction:**

Approximately 42,000 new cases of colorectal cancer (CRC) are diagnosed annually in the United Kingdom with 16,800 deaths. Evidence suggests that quantitative fecal immunochemical tests (FIT) are a good predictor of CRC risk in symptomatic patients presenting to primary care. We aimed to assess the cost-effectiveness of FIT in this setting, considering capacity constraints and waiting times for subsequent colonoscopy.

**Methods:**

We compared two diagnostic FIT strategies, at various thresholds, in the model: (i) FIT for all patients and (ii) current practice where only low-risk patients received FIT. Patients with positive FIT scores and high-risk patients in current practice received colonoscopy. Diagnostic accuracy evidence from published literature, standard UK cost sources, and other sources were used to estimate health outcomes and costs. Waiting times before colonoscopy were assumed proportional to the numbers referred, with the impact of delayed colonoscopy taken from published models. Savings per quality-adjusted life years (QALYs) lost and incremental net monetary benefit (INMB) were used. Uncertainty was evaluated.

**Results:**

Model results suggested that, compared to current practice, FIT generated a positive INMB for the majority of thresholds assessed (GBP200 [USD254] to GBP350 [USD445] per patient at a willingness to pay of GBP20,000 [USD25,474] per QALY gained). A reduction in the number of patients sent to colonoscopy led to cost savings. However, these thresholds were associated with slight QALY losses due to a small proportion of false negative results associated with significantly delayed diagnosis, which outweighed the benefits associated with quicker times to colonoscopy for those with positive FIT results. Savings of over GBP100,000 (USD127,374) per QALY lost were generated. Conclusions were robust to the sensitivity analyses undertaken.

**Conclusions:**

With capacity constraints explicitly represented in the economic modeling, offering FIT to all patients presenting to primary care with symptoms suggestive of CRC was cost effective when compared to current practice. However, the optimal threshold could not be robustly determined due to limited diagnostic accuracy data, parameter uncertainty, and limitations in the model structure; additional primary research could reduce uncertainty.

